# Phosphate binders affect vitamin K concentration by undesired binding, an in vitro study

**DOI:** 10.1186/s12882-017-0560-3

**Published:** 2017-05-02

**Authors:** A. Neradova, S. P. Schumacher, I. Hubeek, P. Lux, L. J. Schurgers, M. G. Vervloet

**Affiliations:** 1 Department of Nephrology, VU University Medical Center, De Boelelaan 1117, 1081 HV Amsterdam, The Netherlands; 2 Department of cardiology, VU University Medical Center, Amsterdam, The Netherlands; 3 Clinical chemistry laboratory, VU University Medical Center Amsterdam, Amsterdam, The Netherlands; 4 Department of Biochemistry, Cardiovascular Research institute Maastricht, University Maastricht, Maastricht, The Netherlands; 5 Institute for Cardiovascular Research VU, ICaR-VU, Amsterdam, The Netherlands

**Keywords:** Phosphate binders, Phosphate, Vitamin K2 binding, In vitro

## Abstract

**Background:**

Vascular calcification is a major contributing factor to mortality in end stage renal disease (ESRD). Despite the efficacy of phosphate binders to improve hyperphosphatemia, data on vascular calcification are less clear. There seems to be a difference in attenuation or delay in progression between different binders. In this in vitro experiment we tested whether phosphate binders could limit bioavailability of vitamin K2 by undesired binding. Vitamin K-deficiency limits activation of the vascular tissue mineralization inhibitor matrix γ-carboxyglutamate (Gla) protein (MGP) thereby exacerbating vascular calcification.

**Methods:**

In this experiment vitamin K2 (menaquinone-7; MK-7) binding was assessed by adding 1 mg of vitamin K2 to a medium with pH 6 containing 67 mg phosphate binder with either 7 mg of phosphate or no phosphate. Five different phosphate binders were tested. After five and a half hours vitamin K was analyzed by HPLC. All experiments were performed in triplicate.

**Results:**

Sucroferric-oxyhydroxide and sevelamer carbonate did not significantly bind vitamin K2, both in solution only containing vitamin K2 or in combination with phosphate. Calcium acetate/magnesium carbonate binds vitamin K2 strongly both in absence (*p* = 0.001) and presence of phosphate (*p* = 0.003). Lanthanum carbonate significantly binds vitamin K2 in solution containing only vitamin K2 (*p* = 0.005) whereas no significant binding of vitamin K2 was observed in the solution containing vitamin K2 and phosphate (*p* = 0.462). Calcium carbonate binds vitamin K2 significantly in a solution with vitamin K2 and phosphate (*p* = 0.009) whereas without phosphate no significant binding of vitamin K2 was observed (*p* = 0.123).

**Conclusions:**

Sucroferric-oxyhydroxide and sevelamer carbonate were the only binders of the five binders studied that did not bind vitamin K2 in vitro. The presence or absence of phosphate significantly interferes with vitamin K2 binding so phosphate binders could potentially limit bioavailability vitamin K2.

## Background

Chronic kidney disease (CKD) is a major and independent risk factor for cardiovascular disease (CVD) [1]. During CKD disease progression, mortality risk due to CVD rises progressively and accounts for 50% of all deaths in the CKD setting [2]. Vascular calcification (VC), almost universally present in late stage CKD, is an important contributor to CVD and CKD-related mortality [3]. A very recent study supports the assumption that density, besides the volume of calcification is an independent risk factor for dismal outcome [4]. Hyperphosphatemia is associated with VC [5]. Despite the widespread use of phosphate binders to control serumphosphate, evidence from prospective trials showing improved outcome by the use of phosphate binders is lacking. In fact it is unknown if the use of phosphate binders, especially calcium based binders, may actually be harmful [6]. There is some attenuation or delay of progression of VC by the non-calcium-based phosphate binders as compared to the calcium containing binders [7–9]. However VC frequently progresses despite adequate phosphate concentrations [10]. This has been attributed to increasing calcium load from calcium based binders [11], chronic inflammation [12] and other factors such as vitamin K deficiency [13]. The prevalence of vitamin K deficiency is high in ESRD patients [14]. Adequate levels of vitamin K are mandatory for activation of matrix γ-carboxyglutamate (Gla) protein (MGP) [15]. MGP is secreted by chondrocytes and vascular smooth muscle cells in the arterial medial layer. MGP requires posttranslational modification by carboxylation and phosphorylation to become a functional calcification inhibitor of VC. We hypothesized that phosphate binders inhibit gastrointestinal uptake of vitamin K2 by undesired binding since the binding of fat soluble vitamins has been described previously [16, 17]. If confirmed this could contribute to vitamin K deficiency and as such possibly to the progress of vascular calcification.

## Methods

### Setup

In an in vitro setup, vitamin K2 (synthetic menaquinone-7; MK-7, kindly provided by Nattopharma ASA, Hovik, Norway), was mixed with several contemporary available phosphate binders, in presence or absence of phosphate, and incubated at pH 6 and fixed temperature of 37 degrees Celsius. Vitamin K2 levels were measured after 330 min. All experiments were performed in triplicate. The tubes, wrapped in aluminum foil, contained a stirring bar and were placed in a glass tank filled with water, which was placed on a magnet stirrer, type RET-GS of IKAR Lab technik and the temperature was kept at 37° by an immersion circulator model 1112A of VWR International BV. The stirring bar allowed continuous stirring of the solution and the heating kept the temperature at 37 degrees Celcius measured by an Easy-read^R^ alcohol based thermometer in a similar tube filled with a stirring bar and the same amount of the same phosphate solution. After 330 min, 1 sample of 4 ml was taken out of each tube, initially containing 148 mcg vitamin K2 (37 mcg/ml solution). Each sample was put in a Rotanta 46R centrifuge of Hettich Zentrifugen for 5 min at 1800 rates per minutes and 20 degrees Celsius and after that 3 ml of supernatant was used for analysis.

### Vitamin K measurement

HPLC, C-18 reversed phase column and fluorometric after post column electrochemical reduction was used to determine the free vitamin K2 (MK-7) concentration. This method does not detect bound vitamin K2. The inter-run variations as reported before were between 6 and 8% [18].

### Materials

Phosphate binders used are shown in Table 1. The abbrevations used are calcium carbonate (CC) lanthanum carbonatehydrate (LA) calcium acetate/magnesium carbonate (CA/MC), sucroferric-oxyhydroxide (SOH) and sevelamer carbonate (SC). Ammonium phosphate from Sigma-Aldrich Co. (St. Louis, USA) was used for phosphate solutions. Ammonia 25% and hydrochloric acid 37% were purchased from Merck (Darmstadt, Germany) and BDH (Fontenay-sous-Bois, France) respectively and used to modulate pH of the medium. A Purelab Flex water purification device of Elga Labwater Global Operations/Veolia Water Solutions & Technologies (United Kingdom) provided purified water for the phosphate solutions and pH media.

**Table 1:** Different phosphate binders, manufacturer and the city and country of origin

Phosphate binder	Manufacturer	City, Country
Lanthanum carbonatehydrate (FosrenolR 750 mg sachets)	Shire Pharmaceutical	Basingstoke, United Kingdom
Calcium acetate/magnesium carbonate (OsvarenR 435 mg/235 mg film-coated tablets)	Fresenius Medical Care Nephrologica	Bad Homburg, Germany
Sevelamer carbonate (RenvelaR 2,4 g sachets)	Sanofi Europe B.V.	Naarden, Netherlands
Calcium carbonate (500 mg chewing tablets)	Fagron BV	Uitgeest, Netherlands
sucroferric-oxyhydroxide (VelphoroR 500 mg chewing tablets)	Vifor Fresenius Medical Care; Renal Pharma France	Neuilly-sur-Seine, France

### Preparation of phosphate binders, phosphate solution, vitamin K2 (MK-7) and pH media

It is not well established what amount of different binders in in vitro setup represent comparable phosphate binding capacity in vivo. The amounts of phosphate binders and phosphate solution were based on half the dosage, which has been used in previous in vitro experiments without vitamin K2 [19]. Therefore, all substances were used in an equal amount of active compound on weight base, and in powder form. CC, CA/MC and SOH tablets were crushed with a pestle. We measured weight and used this value to calculate the amount of supplement needed to get 67 mg of the active compound, showed in formula 1. The concentration of the active compound of the binders was 2.5 mg/ml. The needed amount of supplement was measured by a Mettler AT250 microbalance, with maximum deviation of 0.05 mg.

$$ \mathrm{W} = \left(\mathrm{A}/\mathrm{P}\right) \times 67 $$

Formula 1: W = used amount of substance (mg); A = average weight (mg) of the whole supplement calculated after 10 measurements; *P* = amount of active component of substance (mg) mentioned on the package

Two solutions were prepared pH 6 at start of the experiment. One contained 25 ml of purified water and 1 ml of isopropanol in which 1000 mcg vitamin K2 was dissolved. Concentration of vitamin K2 was 37 mcg/ml. The second solution in addition contained 2.5 mM phosphate to test potential competition of binding. The vitamin K2 content was also measured in solutions containing only a phosphate binder or a phosphate binder with phosphate to exclude interference of the matrix on HPLC with the measuring apparatus.

### Data analysis

Statistical analysis was performed using SPSS 23. An independent sample *T*-test was used to compare the mean values of measured vitamin K2 concentrations.

## Results

All phosphate binders, with the exception of SOH did bind vitamin K in the absence of phosphate. This decline of free K2 concentration was statistically significant for LA and CA/MC. In the presence of phosphate, K2 binding was attenuated for SC and LA, but increased for CC. Table 2 depicts the vitamin K2 concentrations with only vitamin K2 and a phosphate binder in the solution. There is significant binding of vitamin K2 to LA and CA/MC. Vitamin K2 concentrations in a solution containing vitamin K2, phosphate and a binder are shown in Table 3. There is significant binding of vitamin K2 to CA/MC and CC. Vitamin K2 concentrations were also measured in all solutions that did not contain vitamin K2 as a negative control. In none of these samples vitamin K2 was detected. Solutions containing only phosphate with or without a binder only showed negligible vitamin K2 concentration, excluding interference of phosphate or phosphate binders with the vitamin K2 measurement (data not shown).

**Table 2:** Vitamin K2 concentration in solution with vitamin K2 and phosphate binders

	Vitamin K2 concentration mcg/ml (SD)	*P*-value compared to control
control	34.3 (4.35)	
CC	25.9 (5.86)	0.123
LA	<0.001^a^ (<0.001)	0.005
CA/MC	6.47 (3.51)	0.001
SOH	47.27 (8.71)	0.106
SC	19.79 (11.43)	0.147

First column depicts the mean values of measured Vitamin K2 concentrations in mcg/ml in a solution containing only vitamin K2 and a phosphate binder. *P* value is the comparison between control and phosphate binder. *CC* Calcium carbonate, *LA* Lanthanum carbonatehydrate, *CA/MC* calcium acetate/magnesium carbonate, *SOH* sucroferric-oxyhydroxide and *SC* sevelamer carbonate. ^a^ this value represents a figure below the detection limit, which was 40 pg/ml

**Table 3:** Vitamin K2 concentration is solution with vitamin K2, phosphate and phosphate binders

	Vitamin K2 concentration mcg/ml (SD)	*P*-value compared to control
control	27.53 (4.89)	
CC	0.497 (0.86)	0.009
LA	21.15 (11.94)	0.462
CA/MC	1.84 (3.19)	0.003
SOH	41.8 (10.80)	0.135
SC	32.9 (11.91)	0.528

First column depicts the mean values of measured Vitamin K2 concentrations in mcg/ml in a solution containing vitamin K2, phosphate and a phosphate binder. *P* value is the comparison between control and phosphate binder. *CC* Calcium carbonate, *LA* Lanthanum carbonatehydrate, *CA/MC* calcium acetate/magnesium carbonate, *SOH* sucroferric-oxyhydroxide and *SC* sevelamer carbonate

## Discussion

In this in vitro study we demonstrate that calcium-containing phosphate binders and lanthanum carbonate bind vitamin K2. For LA this binding depends on the absence of phosphate, pointing to competitive binding between phosphate and vitamin K2 for this compound. For calcium carbonate this vitamin K2 binding was statistically significant in the presence of phosphate compared to the solution without phosphate. In the mixture with sevelamer carbonate a nominally lower concentration of K2 was shown as well, but this decline was not statistically significant. Addition of sucroferric- oxyhydroxide did not lead to any decline of vitamin K2 at all, irrespective of presence or absence of phosphate. Our study design precludes a formal quantitative comparison between the different phosphate binders. Several remarkable differences however appear obvious. First, both calcium containing compounds had increased affinity for vitamin K2 in the presence of phosphate. We did not study the chemical explanation for this feature, but possibly the formed calciumphosphate salt itself binds K2 as well. For lanthanum carbonate there appears to be competition for binding between phosphate and vitamin K2. Although we could not find a statistically significant amount of vitamin K2 binding by sevelamer carbonate, where a previous study did [17], we did observe a trend in the same direction. This may be due to difference in concentrations used: 2.5 mg/ml and 4 mg/ml for sevelamer cabonate; and 37 mcg/ml (our study) and 0.5 and 5 ug/ml (Takagi et al.) for vitamin K. Moreover the previous research did not report whether vitamin K1 or K2 was used as substrate [17]. SOH showed no vitamin K2 binding at all. The statistically non-significant higher values of vitamin K2 when using the latter binder, as compared to control is probably the consequence of assay variability. Extrapolating these results to biological systems, including the treatment of hyperphosphatemia in CKD patients can only be done with great caution. However, our data do point to a feature of some commonly used phosphate binders that may have clinical consequences. Vitamin K deficiency results in undercarboxylation of MGP, and as such limit natural defense against ectopic calcification including VC. To some extent, the enigmatic lack of improvement of relevant endpoint in clinical studies, despite effective phosphate lowering potential, could be attributed to a decline in bioavailability of vitamin K from the gastrointestinal tract by phosphate binder therapy. In recent years some studies suggested superior clinical outcome when using sevelamer carbonate as compared to calcium containing binders [20, 21]. Generally this has been ascribed to the calcium loading as a consequence of calcium content in these binders. Our data suggest an additional mechanistic explanation, which is the increased likelihood of vitamin K deficiency. In support of this hypothesis is also a study in which non-hyperphosphatemic CKD patients were treated with either placebo or phosphate binder therapy. Despite a small decline in serum phosphate there was an increase in arterial calcification in the active treatment arm [10]. Our finding that vitamin K2 binding by LA is absent in presence of phosphate does not necessarily imply that in patients no relevant vitamin K binding occurs. It is likely that high gastrointestinal levels of phosphate do not always match local high concentrations of phosphate binders. In addition, dose up titration in case of persistent hyperphosphatemia may increase likelihood of unnoticed aspecific binding of non-phosphorus compounds. This may especially apply in hyperparathyroidism where a substantial amount of phosphate may be bone-derived. Our study has some limitations. We used a fixed pH which does not necessarily match the gastrointestinal tract, where pH may be as low as 1 in the stomach. However, at sites where vitamin K2 uptake occurs pH is not low, so our set-up may be more relevant than at low pH. A single measurement of vitamin K2 was performed at 330 min which was considered a reasonable period to mimic the time needed for the passage to the terminal ileum where the absorption of vitamin K2 starts [22]. Furthermore, only vitamin K2 has been used in our experiments, since vitamin K2 is the preferred cofactor for the carboxylation of MGP [23]. In addition, phosphate and vitamin K concentrations in vivo may differ substantially, and moreover a wide range of additional aspects may be very different as well. In our study we mainly aimed to test the proof of principle of the existence of vitamin K2 binding by contemporary phosphate binders. Obviously, the clinical relevance of our findings cannot be assessed in an in vitro setup. Another limitation, as described, is the fact that no quantitative comparison between phosphate binders can be made.

## Conclusion

Sucroferric-oxyhydroxide and sevelamer carbonate were the only binders of the five binders studied that did not bind vitamin K2 in vitro. The clinical significance of this finding requires additional studies.

## Abbreviations

CA/MC: Calcium acetate/magnesium carbonate
CC: Calcium carbonate
CKD: Chronic kidney disease
CVD: Cardiovascular disease
LA: Lanthanum carbonatehydrate
MK-7: Synthetic menaquinone-7
SC: Sevelamer carbonate
SOH: Sucroferric-oxyhydroxide
VC: Vascular calcification
